# Erythematous capillary-lymphatic malformations mimicking blood vascular anomalies

**DOI:** 10.1172/jci.insight.172179

**Published:** 2023-10-23

**Authors:** René Hägerling, Malou Van Zanten, Rose Yinghan Behncke, Sascha Ulferts, Nils R. Hansmeier, Bruno Märkl, Christian Witzel, Bernard Ho, Vaughan Keeley, Katie Riches, Sahar Mansour, Kristiana Gordon, Pia Ostergaard, Peter S. Mortimer

**Affiliations:** 1 Institute of Medical and Human Genetics, Charité – Universitätsmedizin Berlin, Corporate Member of Freie Universität Berlin and Humboldt-Universität zu Berlin, Berlin, Germany.; 2 Berlin Institute of Health at Charité – Universitätsmedizin Berlin, BIH Center for Regenerative Therapies, Berlin, Germany.; 3 Berlin Institute of Health at Charité – Universitätsmedizin Berlin, BIH Academy, Clinician Scientist Program, Berlin, Germany.; 4 Research Group Development and Disease, Max Planck Institute for Molecular Genetics, Berlin, Germany.; 5 Molecular and Clinical Sciences Institute, St George’s University of London, London, United Kingdom.; 6 Dermatology and Lymphovascular Medicine, St George’s University Hospitals NHS Foundation Trust, London, United Kingdom.; 7 Institute of Pathology and Molecular Diagnostics, University Clinic Augsburg, Augsburg, Germany.; 8 Department of Surgery, Campus Charité Mitte and Campus Virchow-Klinikum, Charité – Universitätsmedizin Berlin, Corporate Member of Freie Universität Berlin and Humboldt-Universität zu Berlin, Berlin, Germany.; 9 Lymphoedema Clinic, Derby Hospitals Foundation NHS Trust, Derby, United Kingdom.; 10 SW Thames Regional Centre for Genomics, St George’s University Hospitals NHS Foundation Trust, London, United Kingdom.

**Keywords:** Angiogenesis, Cell Biology, Diagnostic imaging, Lymph

## Abstract

Superficial erythematous cutaneous vascular malformations are assumed to be blood vascular in origin, but cutaneous lymphatic malformations can contain blood and appear red. Management may be different and so an accurate diagnosis is important. Cutaneous malformations were investigated through 2D histology and 3D whole-mount histology. Two lesions were clinically considered as port-wine birthmarks and another 3 lesions as erythematous telangiectasias. The aims were (i) to demonstrate that cutaneous erythematous malformations including telangiectasia can represent a lymphatic phenotype, (ii) to determine if lesions represent expanded but otherwise normal or malformed lymphatics, and (iii) to determine if the presence of erythrocytes explained the red color. Microscopy revealed all lesions as lymphatic structures. Port-wine birthmarks proved to be cystic lesions, with nonuniform lymphatic marker expression and a disconnected lymphatic network suggesting a lymphatic malformation. Erythematous telangiectasias represented expanded but nonmalformed lymphatics. Blood within lymphatics appeared to explain the color. Blood-lymphatic shunts could be detected in the erythematous telangiectasia. In conclusion, erythematous cutaneous capillary lesions may be lymphatic in origin but clinically indistinguishable from blood vascular malformations. Biopsy is advised for correct phenotyping and management. Erythrocytes are the likely explanation for color accessing lymphatics through lympho-venous shunts.

## Graphical abstract

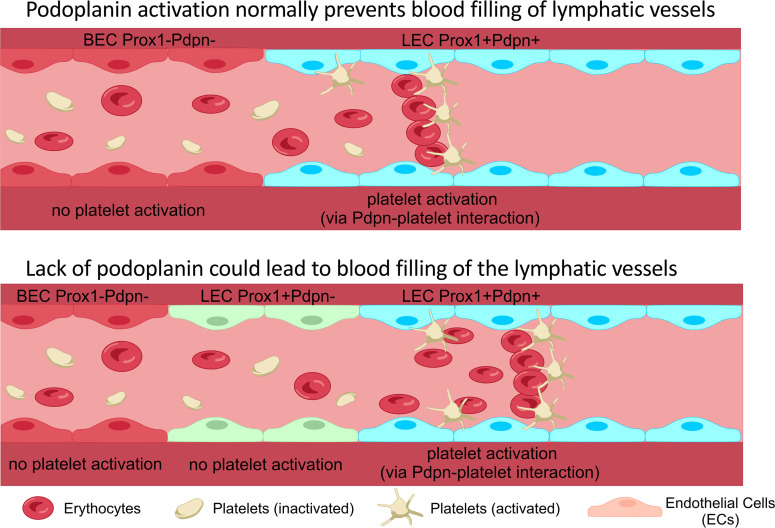

## Introduction

A general assumption is that an erythematous cutaneous vascular malformation (nevus) is a disorder of dermal blood vessels. Rarely is consideration given to the possibility that this may represent a lymphatic structure. This may have implications for phenotyping and management.

In his 2015 classification of capillary malformations, Happle makes no mention of cutaneous lymphatic malformations that may be red. The assumption would be that they are blood vascular malformations (1). In the clinic, differentiation may be relatively straightforward in a classic lymphangioma circumscriptum containing blood but not so in other cutaneous lymphatic malformations that are red.

In the International Society for the Study of Vascular Anomalies (ISSVA) classification of capillary malformations, no consideration is given to the fact that cutaneous erythematous nevi or erythematous cutaneous telangiectasias may be lymphatic in origin, nor in the classification of lymphatic malformations is mention made of cutaneous involvement as a red birthmark (2).

We describe 5 cases, 2 displaying port-wine birthmarks (or nevus flammeus) and 3 exhibiting erythematous cutaneous telangiectasias, where the clinical diagnosis was a (blood) capillary malformation, but all proved with histological analysis to be a lymphatic vessel structure.

The purpose of the report is 1) to determine if cutaneous erythematous malformations including port-wine birthmarks and telangiectasias can represent a lymphatic phenotype, 2) to determine if the lesions represent expanded but otherwise normal dermal lymphatic vessels or malformed lymphatics, and 3) to determine if the presence of red blood cells was the explanation for the red color.

For this purpose, we have performed classical 2-dimensional (2D) histological analysis as well as whole-mount 3-dimensional (3D) histology. In contrast to physical sectioning in 2D histology approaches, 3D histology represents a light sheet imaging–based optical-sectioning methodology, which allows generation of series of optical sections from immunofluorescence-stained, optically cleared tissue samples (3–5). Following digital 3D reconstruction of the optical sections, the entire lymphatic vascular network is visualized in 3D space. Therefore, 3D histology represents a brilliant tool for vascular phenotyping and in-depth understanding of the underlying vascular alterations.

## Results

Presented here are 5 cases of erythematous cutaneous capillary malformations that clinically would be described as birthmarks or nevi. Two cases exhibited port-wine birthmark lesions (cases 1–2), and in 3 cases dark red telangiectasias were observed in the skin of a swollen thigh (cases 3–5) (Table 1).

### Case 1: segmental overgrowth and vascular malformation (Klippel-Trenaunay syndrome) of right hindquarter (nevus flammeus)

**Figure 1:**
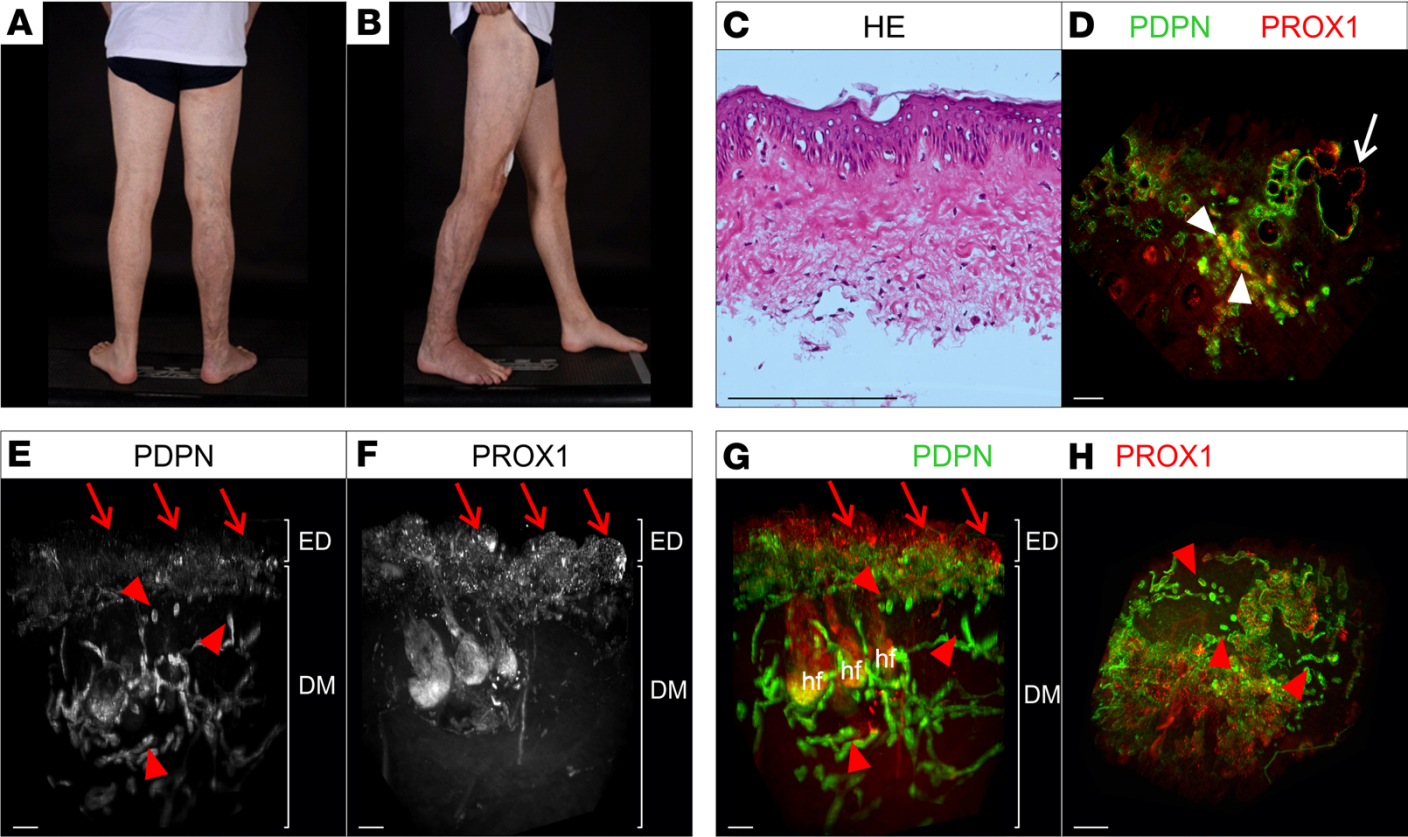
Macroscopic as well as 2-dimensional and 3-dimensional microscopic manifestations of a patient (case 1) with Klippel-Trenaunay syndrome. (**A** and **B**) Patient presenting with Klippel-Trenaunay syndrome (KTS) with extensive venous abnormalities, dusky red port-wine birthmark, and slight lymphedema in the right leg and foot but no overgrowth. (**C**) Standard histological analysis of a skin biopsy from the area of the port-wine birthmark using hematoxylin and eosin (H&E) stain of microtome sections. (**D**–**H**) 2D optical section (**D**) as well as 3D reconstruction (**E**–**H**) of lymphatic vessels of whole-mount immunostained affected patient tissue (port-wine birthmark) imaged using light sheet microscopy. Podoplanin (PDPN) served as a lymphatic endothelial cell surface marker and the transcription factor Prospero-related homeobox 1 (PROX1) as a lymphatic endothelial nuclei marker. Detected antigens and respective colors are indicated. (**D**) Representative 2D optical sections of whole-mount immunostained affected patient tissue. Blood-filled lymphatic vessels are marked by white arrowheads. PDPN-negative, PROX1-positive vessels are marked by white arrow. (**E**–**G**) Maximum-intensity projections of 3D reconstructed lymphatic vasculature. Visualization of the tissue volume with the epidermis (ED) apically and the papillary dermis located at top and cutaneous plexus at bottom of the dermis (DM). PDPN-negative, PROX1-positive cystic vascular lesions located underneath the epidermis are highlighted using red arrows. Red arrowheads: fragmented vessels. (**H**) Digitally rotated view of the same specimen, showing the vessels of the papillary plexus viewed en face through the epidermis. Red arrowheads: fragmented vessels. Scale bars: 200 μm. hf, hair follicle.

A 24-year-old man was referred for management of swelling of his right leg. He was noted to have obvious varicose veins as well as a port-wine birthmark/nevus flammeus extending up the entire right leg from ankle to groin, which had been present since birth. There was pitting edema detectable in the right leg and foot but not the left leg, which was normal. There was no limb length discrepancy, although his right leg was slightly bigger in girth than the left leg. He had been diagnosed with Klippel-Trenaunay syndrome (KTS) (Figure 1, A and B). Lymphoscintigraphy showed mild abnormalities of lymphatic function. A skin biopsy was obtained from the affected leg. No postzygotic mosaic pathogenic variants were detected in the phosphatidylinositol 3-kinase catalytic subunit alpha (*PIK3CA*) gene, nor were any of the genes in the AKT pathway or the RAS/MAP kinase pathway.

#### 2D histology.

Hematoxylin and eosin (H&E) staining of the specimen revealed no obvious lymphatic or blood vascular alteration (Figure 1C). Immunofluorescence staining of sections for the pan-endothelial marker CD31 as well as the lymphatic vessel marker Podoplanin showed presence of lymphatic and blood vessels but did not provide any further information on a possible lymphatic phenotype or the origin of the red color of the lesion (Supplemental Figure 1, A–D; supplemental material available online with this article; https://doi.org/10.1172/jci.insight.172179DS1).

#### Whole-mount 3D histology.

Analysis of the acquired 2D optical sections of the affected tissue biopsy showed dilated, vascular cystic lesions in the papillary dermis (Figure 1D). The cystic lesions showed strong expression of the lymphatic markers Prospero-related homeobox 1 (PROX1) and Podoplanin, indicating a lymphatic vascular origin of the nevus flammeus. However, further detailed examination of the expression of PROX1 and Podoplanin in all optical sections revealed a nonuniform expression of Podoplanin, whereas PROX1 expression was unaltered (Figure 1D, white arrow). In addition, red blood cells were detected within PROX1-positive, Podoplanin-positive vessels using autofluorescence (Figure 1D, white arrowheads).

The 3D reconstruction of the entire biopsy provided additional information on the lymphatic phenotype. In contrast with the area in the papillary dermis showing Podoplanin-negative, PROX1-positive lesions (Figure 1, E–G, red arrows), deeper lymphatic vessels in the dermis showed no presence of cystic lymphatic lesions and only few dilated lymphatic vessels. In the lymphatic vasculature of the deeper dermis, no lymphatic valves were detected (data not shown). Nonconnected lumenized lymphatic vessels were detected in the dermis (Figure 1, E–H, red arrowheads). En face view of the lymphatic cystic lesions directly underneath the epidermis showed the nonuniformly distributed presence of microcysts (Figure 1H).

### Case 2: segmental overgrowth and vascular malformation of the left forequarter (nevus flammeus)

**Figure 2:**
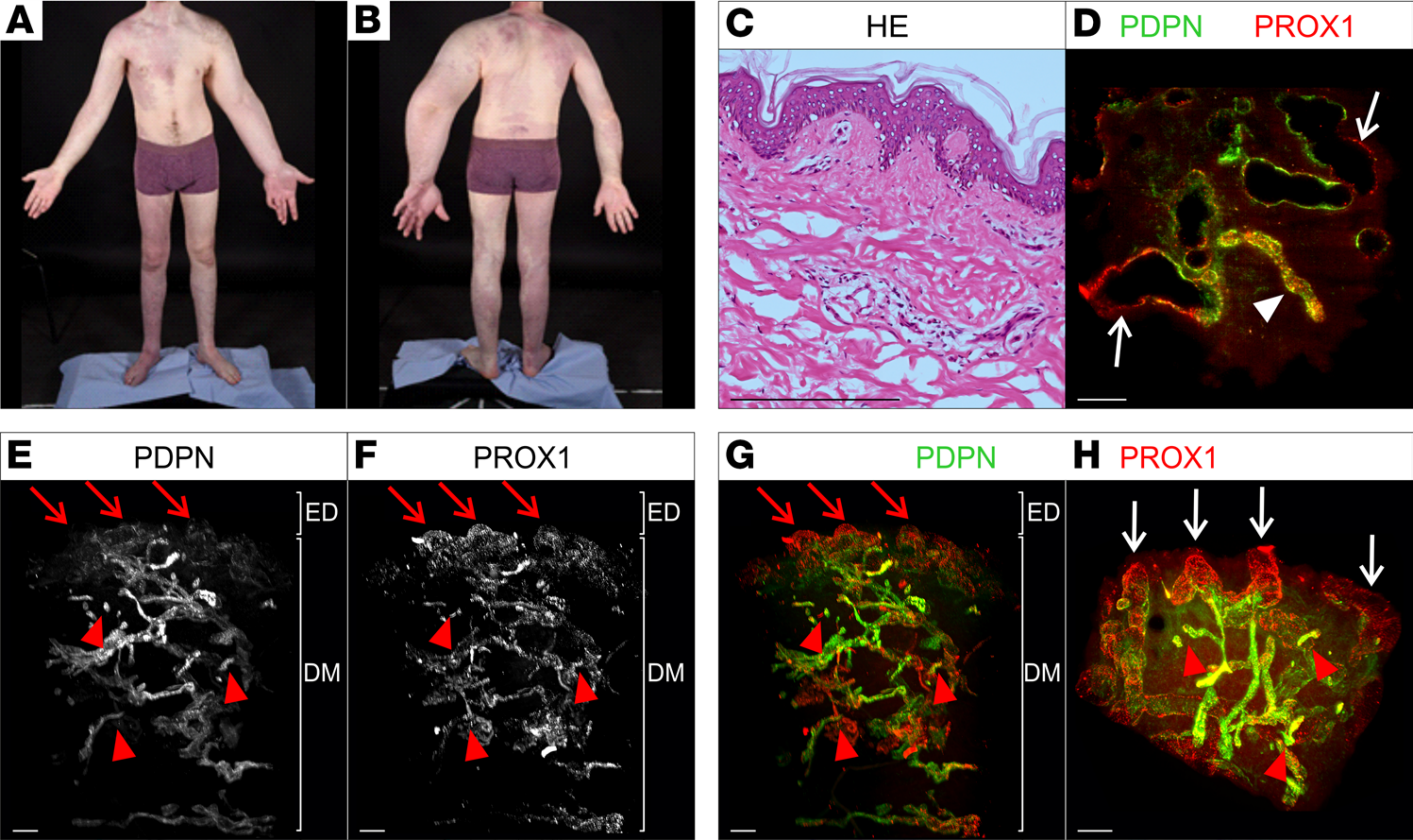
Macroscopic as well as 2D and 3D microscopic manifestations of a patient (case 2) with KTS and port-wine birthmark. (**A** and **B**) Clinical manifestations of patient with KTS presenting with extensive port-wine birthmarks associated with segmental overgrowth, scoliosis, venous disease, and foot swelling. (**C**) Standard histological analysis of a skin biopsy with port-wine birthmark using hematoxylin and eosin stain of microtome sections. (**D**–**H**) 2D optical section (**D**) as well as 3D reconstruction (**E**–**H**) of lymphatic vessels of whole-mount immunostained affected patient tissue (port-wine birthmark) imaged using light sheet microscopy. Podoplanin (PDPN) served as a lymphatic endothelial cell membrane marker and the transcription factor PROX1 as a lymphatic endothelial nuclei marker. Detected antigens and respective colors are indicated. (**D**) Representative 2D optical sections of whole-mount immunostained affected patient tissue. Blood-filled lymphatic vessel is marked by white arrowhead. Dilated PDPN-negative, PROX1-positive vessels are marked by white arrows. (**E**–**G**) Maximum-intensity projections of 3D reconstructed lymphatic vasculature. Visualization of the tissue volume with the papillary dermis located at top and cutaneous plexus at bottom of dermis (DM). PDPN-negative, PROX1-positive cystic vascular lesions located underneath the epidermis (ED) are highlighted using red arrows. Red arrowheads: fragmented vessels. (**H**) Digitally rotated view of the same specimen, showing the vessels of the papillary plexus viewed en face through the epidermis. White arrows: PROX1-positive, PDPN-negative cystic vascular lesions. Red arrowheads: fragmented vessels. Scale bars: 200 μm.

A 24-year-old man had lymphedema and overgrowth of his left upper limb and scapula from birth. Extensive port-wine birthmarks were present on both legs, upper torso, right upper arm, and neck. There was no segmental overgrowth of the lower limbs, but there were extensive venous varicosities and engorgement (Figure 2, A and B). The right foot was slightly swollen with 2-3 syndactyly. On venous duplex ultrasound there was incompetence of deep veins, posterior tibial vein, and peroneal vein as well as the long saphenous and perforating veins seen in the right leg. In the left leg, there was incompetence of the short saphenous and perforating veins only. DNA was extracted from a skin biopsy of the affected limb. No postzygotic mosaic pathogenic variants were detected in the *PIK3CA* gene, any of the genes in the AKT pathway, or any in the RAS/MAP kinase pathway.

#### 2D histology.

No discrimination between lymphatic and blood vessel phenotype could be detected in H&E staining (Figure 2C). The immunofluorescence staining for blood (CD31) and lymphatic vessel markers (Podoplanin) showed presence of lymphatic vessels and normal blood vessels. No erythrocytes could be detected within lymphatic vessels (Supplemental Figure 1, E–H).

#### Whole-mount 3D histology.

Analysis of the optical sections revealed the presence of dilated, hyperplastic lymphatic vessels located in the papillary dermis (Figure 2D). In accordance with the findings in Figure 1D, a nonuniform expression of Podoplanin in the vessels was detected. Strong PROX1 expression was not altered in these vessels (Figure 2D, white arrows). The presence of blood-filled Podoplanin-positive, PROX1-positive vessels was detected using autofluorescence (Figure 2D, white arrowhead).

The 3D reconstruction of the entire biopsy provided additional information on the lymphatic phenotype. In contrast with the area in the papillary dermis showing Podoplanin-negative, PROX1-positive lesions (Figure 1, E–G, red arrows), deeper lymphatic vessels in the dermis showed no presence of cystic lymphatic lesions and only few dilated lymphatic vessels. In the lymphatic vasculature of the deeper dermis, no lymphatic valves were detected. Nonconnected lumenized lymphatic vessels were detected in the dermis (Figure 2, E–H, red arrowheads). En face view of the lymphatic cystic lesions in the papillary dermis showed the nonuniformly distributed presence of microcysts (Figure 2H, white arrows).

### Case 3: WILD syndrome (erythematous telangiectasia)

**Figure 3:**
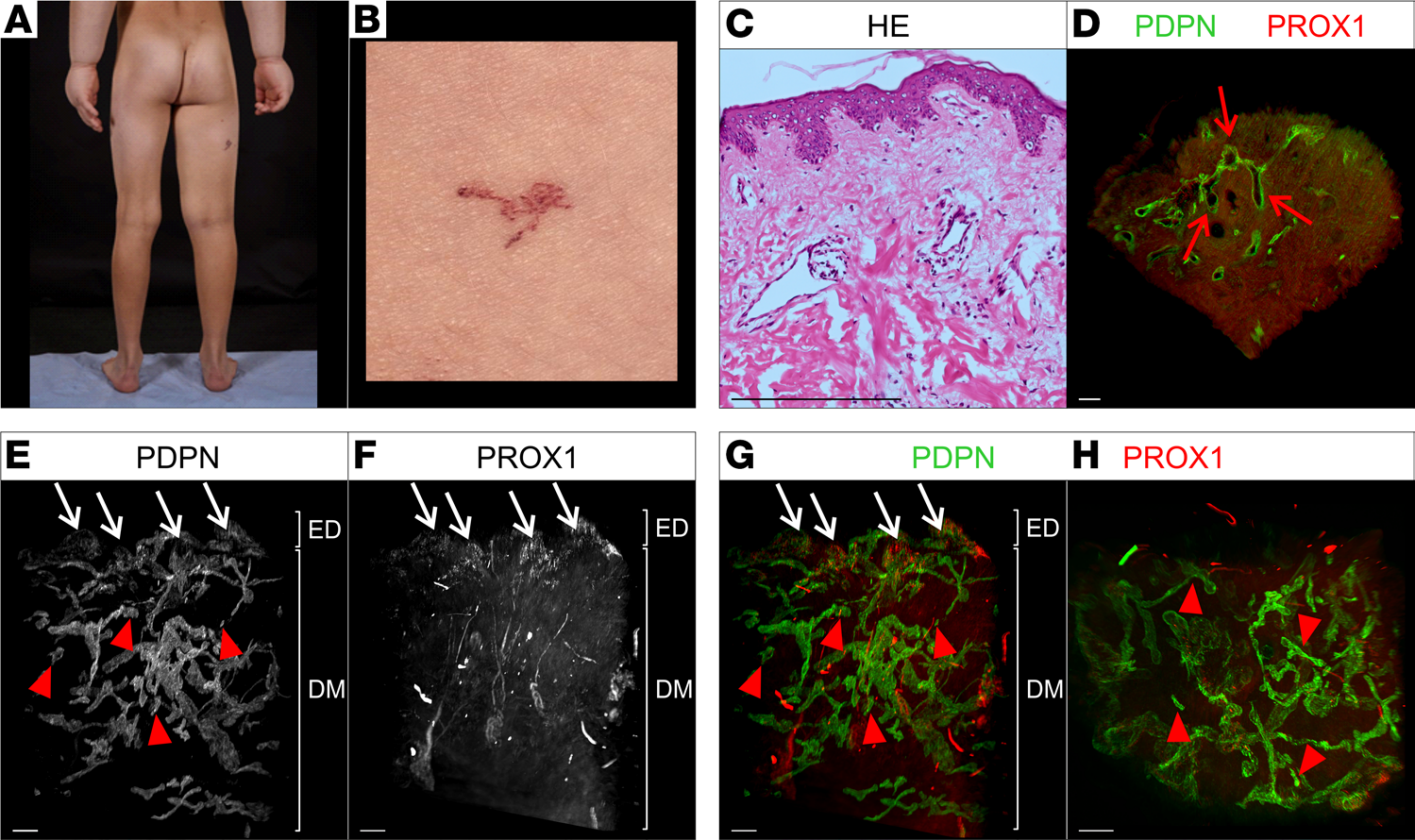
Macroscopic as well as 2D and 3D microscopic manifestations of a patient (case 3) with WILD syndrome, a widespread congenital lymphedema. (**A** and **B**) Clinical manifestations of patient presenting with swollen “boxing glove” hands, right thigh lymphedema, and dark erythematous telangiectasias on back and side of thigh. (**C**) Standard histological analysis of a skin biopsy with telangiectasia using hematoxylin and eosin stain of microtome sections. (**D**–**H**) 2D optical section (**D**) as well as 3D reconstruction (**E**–**H**) of lymphatic vessels of whole-mount immunostained affected patient tissue (telangiectasia) imaged using light sheet microscopy. Podoplanin (PDPN) served as a lymphatic endothelial cell membrane marker and the transcription factor PROX1 as a lymphatic endothelial nuclei marker. Detected antigens and respective colors are indicated. (**D**) Representative 2D optical sections of whole-mount immunostained affected patient tissue. Dilated lymphatic vessels are marked by red arrows. (**E**–**G**) Maximum-intensity projections of 3D reconstructed lymphatic vasculature. Visualization of the tissue volume with the papillary dermis located at top and cutaneous plexus at bottom of dermis (DM). PDPN-negative, PROX1-positive dilated vessels are highlighted using white arrows. Red arrowheads: fragmented vessels. (**H**) Digitally rotated view of the same specimen, showing the vessels of the papillary plexus viewed en face through the epidermis. Red arrowheads: fragmented vessels. Scale bars: 200 μm.

A 11-year-old boy was born with bilateral upper limb primary lymphedema with “boxing glove” swelling of the hands (Figure 3A), right thigh lymphedema, genital lymphedema as well as widespread cutaneous lymph blisters (lymphangiectasia) particularly on the trunk, and scattered red spider-like capillaries also in the skin (Figure 3B). No segmental overgrowth was observed, and no venous problems were reported. He has been given a working diagnosis of WILD syndrome (warts, immunodeficiency, and lymphatic dysplasia) (6, 7). The erythematous telangiectasias appeared evanescent as they could come and go over weeks of observation. One of the erythematous telangiectasia was biopsied. The genetic cause of WILD syndrome has not yet been identified, so whole-genome sequencing (as part of Genomics England’s 100,000 Genomes Project) was performed. No pathogenic variants were identified. Mosaicism is suspected and genetic analysis is being performed on DNA from skin fibroblasts as part of an ongoing research study to identify the cause of WILD syndrome.

#### 2D histology.

H&E staining showed dilated vascular lumens, most likely lymphatic vessels (Figure 3C and Supplemental Figure 1, I–L).

#### Whole-mount 3D histology.

Analysis of the acquired 2D optical sections of the affected tissue biopsy showed dilated weakly PROX1-positive, strongly Podoplanin-positive vessels in the area of the papillary dermis (Figure 3D, red arrows) but no cystic vascular structures. The lymphatic vessel density appeared increased compared with healthy control samples (Supplemental Figure 2). Further detailed examination of the expression of PROX1 and Podoplanin in all optical sections revealed a uniform, nonaltered expression of Podoplanin and PROX1.

The 3D reconstruction of the entire sample provided additional information on lymphatic vessels. In contrast with the lymphatic vasculature in normal skin, the visualized lymphatic vasculature did not show hierarchical organization of the vascular tree but hyperplastic, dilated lymphatic vessels in the deeper dermis (Figure 3, E–G). Nonconnected lumenized vessel fragments were present (Figure 3, E, G, and H, red arrowheads). In contrast with the papillary dermis (Figure 3, E–G, white arrow), PROX1 was expressed only weakly in deeper dermis (Figure 3, F and G). In the lymphatic vasculature of the deeper dermis, no lymphatic valves were detected.

### Case 4: WILD syndrome (erythematous telangiectasia)

**Figure 4:**
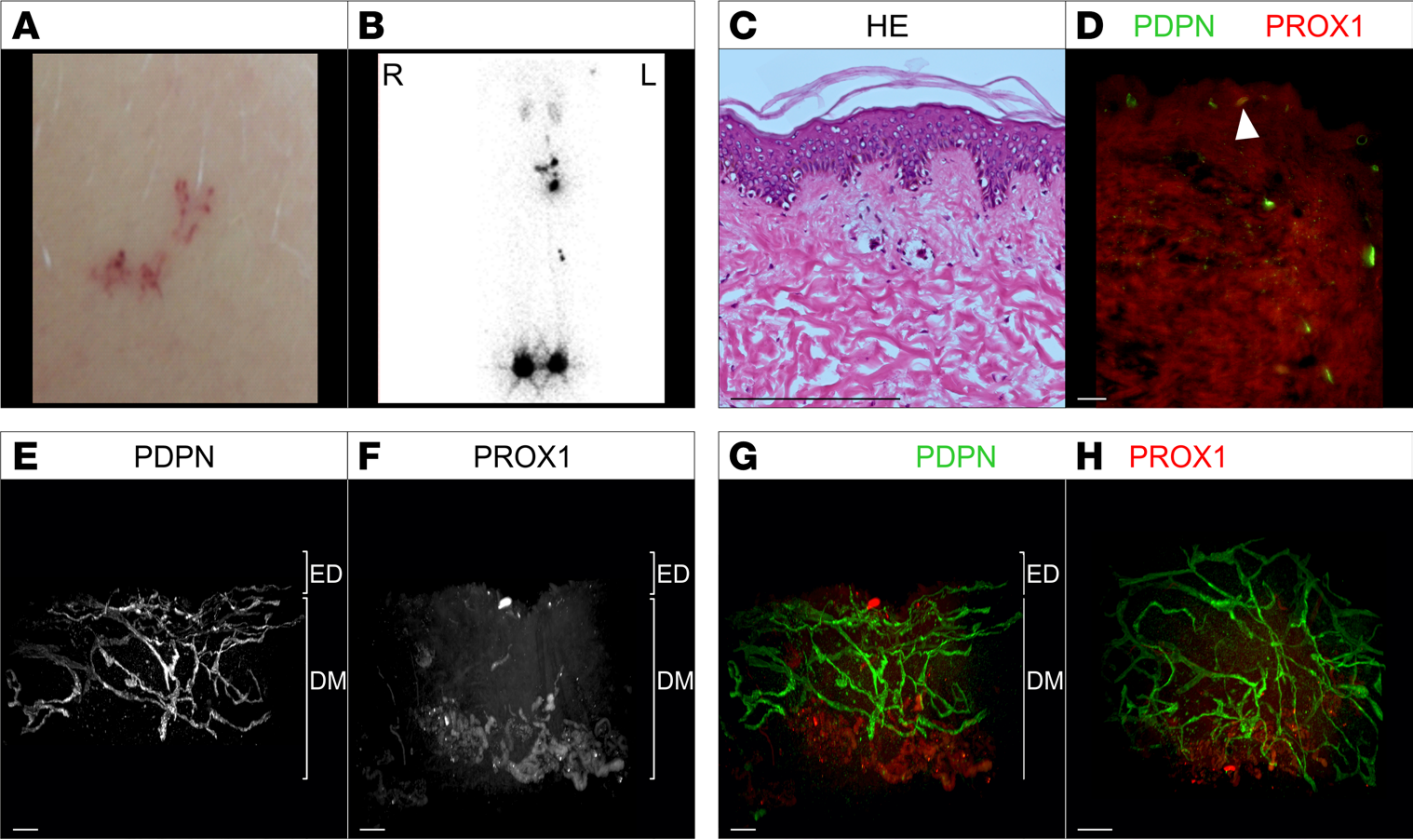
Macroscopic as well as 2D and 3D microscopic manifestations of a patient (case 4) with WILD syndrome and erythematous telangiectasia. (**A**) Telangiectasia in the skin of swollen left thigh. (**B**) Lymphoscintigraphy showing a posterior-anterior image with no visible tracer drainage in the left leg but uptake in the right popliteal nodes, indicating deep lymph drainage, which is an abnormal finding despite a normal leg clinically. (**C**) Standard histological analysis of a skin biopsy with telangiectasia using hematoxylin and eosin stain of microtome sections. (**D**–**H**) 2D optical section (**D**) as well as 3D reconstruction (**E**–**H**) of lymphatic vessels of whole-mount immunostained affected patient tissue (telangiectasia) imaged using light sheet microscopy. Podoplanin (PDPN) served as a lymphatic endothelial cell membrane marker and the transcription factor PROX1 as a lymphatic endothelial nuclei marker. Detected antigens and respective colors are indicated. (**D**) Representative 2D optical sections of whole-mount immunostained affected patient tissue. Blood-filled lymphatic vessels are marked by white arrowhead. (**E**–**G**) Maximum-intensity projections of 3D reconstructed lymphatic vasculature. Visualization of the tissue volume with the papillary dermis located at top and cutaneous plexus at bottom of the dermis (DM). (**H**) Digitally rotated view of the same specimen, showing the vessels of the papillary plexus viewed en face through the epidermis. Scale bars: 200 μm.

A 21-year-old woman presented with pubertal onset of swelling of her left leg, consistent with primary lymphedema. The lymphedema extended into the left flank and buttock, but there was no limb length discrepancy. No segmental overgrowth was observed, and no venous problems were reported. She had hypertrophy/edema of the left breast. She had what was considered a cutaneous vascular malformation on both sides of her neck, and there were 2 small telangiectasias on her left thigh (Figure 4A). Lymphoscintigraphy showed rerouted lymph drainage through the deep lymphatic vessels via popliteal nodes but with normal levels of transport in the nonswollen left leg and functional aplasia in the swollen right leg (Figure 4B). A working diagnosis of WILD syndrome was made (7). Genetic analysis is being performed on DNA from skin fibroblasts as part of an ongoing research study to identify the cause of WILD syndrome.

#### 2D histology.

H&E staining as well as staining for lymphatic vessel markers revealed no obvious vascular alteration (Figure 4C and Supplemental Figure 1, M–P).

#### Whole-mount 3D histology.

In comparison with healthy control (Supplemental Figure 2), 3D histology of the entire lymphatic vasculature as shown in 2D (Figure 4D) as well as 3D (Figure 4, E–H) revealed a normal, nondilated lymphatic vessel architecture and low PROX1 expression. A very low number of valves was detectable compared with control samples (Supplemental Figure 2). Neither cystic vascular lesion nor nonconnected vessel fragments were detected. However, a Podoplanin-positive lymphatic vessel, which is packed with erythrocytes, was seen (Figure 4D, white arrowhead), indicating possible connections between lymph and blood vessels. On closer inspection, erythrocytes, highlighted by autofluorescence, were observed within unstained blood vessels draining into the Podoplanin-positive lymphatic vessel (Supplemental Figure 3). This indicated a potential shunting site, which could not be investigated further with the current material.

### Case 5: WILD syndrome (erythematous telangiectasia)

**Figure 5:**
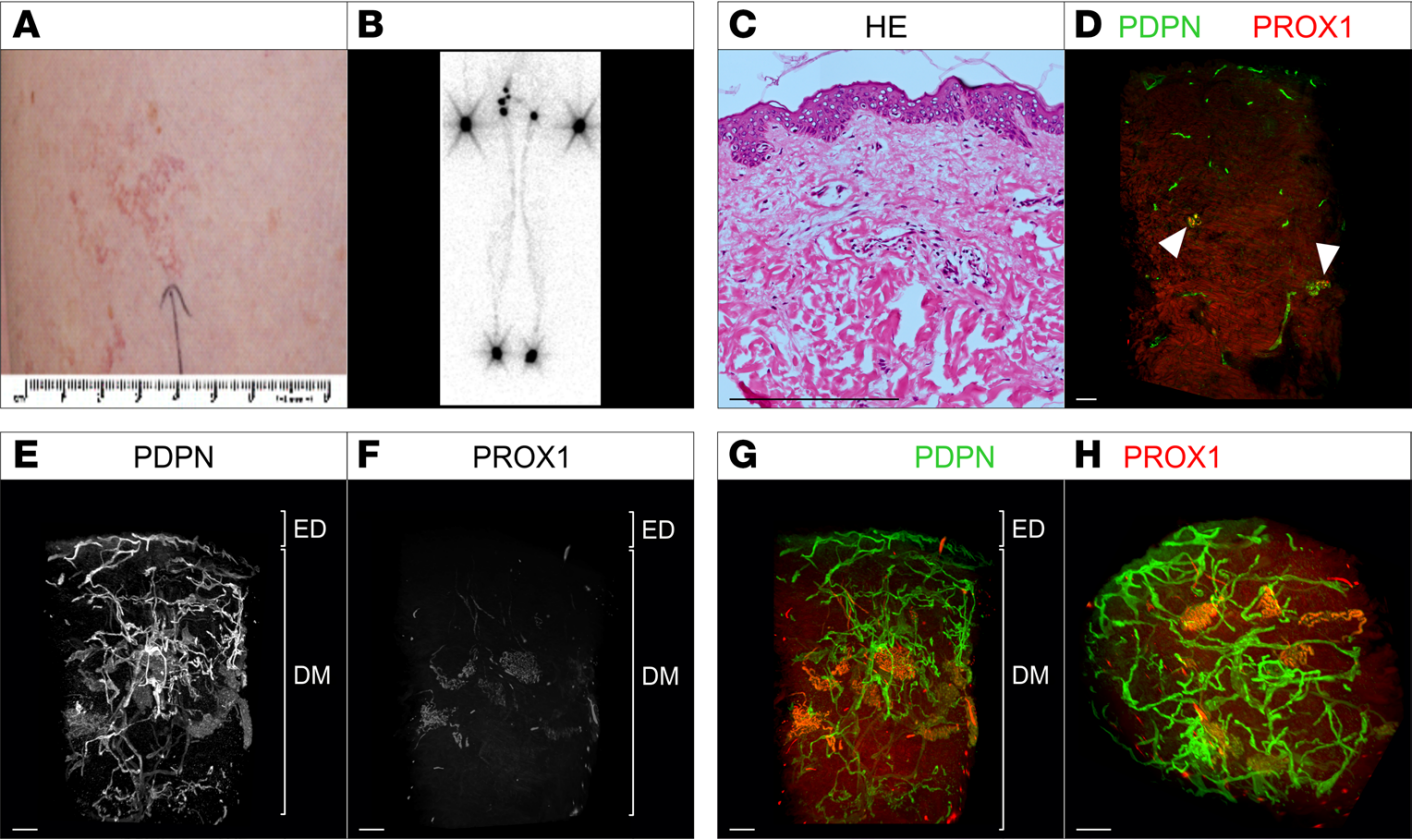
Macroscopic as well as 2D and 3D microscopic manifestations of a patient (case 5) with WILD syndrome and erythematous telangiectasia. (**A**) Clinical manifestations of patient presenting with telangiectasia in skin of left thigh from swollen limb. (**B**) Lymphoscintigraphy showing a posterior-anterior image showing reduced lymph node uptake of tracer in the left groin but otherwise normal-looking lymph drainage pathways in both legs. (**C**) Standard histological analysis of a skin biopsy with telangiectasia using hematoxylin and eosin stain of microtome sections. (**D**–**H**) 2D optical section (**D**) as well as 3D reconstruction (**E**–**H**) of lymphatic vessels of whole-mount immunostained affected patient tissue (telangiectasia) imaged using light sheet microscopy. Podoplanin (PDPN) served as a lymphatic endothelial cell-membrane marker, the transcription factor PROX1 as lymphatic endothelial nuclei marker. Detected antigens and respective colors are indicated. (**D**) Representative 2D optical sections of whole-mount immunostained affected patient tissue. Blood-filled lymphatic vessels are marked by white arrowheads. (**E**–**G**) Maximum-intensity projections of 3D reconstructed lymphatic vasculature. Visualization of the tissue volume with the papillary dermis located at top and cutaneous plexus at bottom of the dermis (DM). (**H**) Digitally rotated view of the same specimen, showing the vessels of the papillary plexus viewed en face through the epidermis. Scale bars: 200 μm.

A 22-year-old woman presented at birth with lymphedema of her left lower limb/hindquarter, left upper limb, and left side of the face. No overgrowth was observed, and no venous problems were reported. Also noted was a cutaneous vascular lesion on the left side of the chest and fixed erythematous telangiectasia on the left thigh (Figure 5A). These abnormalities did not change and had grown with her. Lower limb lymphoscintigraphy revealed reduced lymph node uptake of tracer in the left groin but otherwise normal-looking lymph drainage pathways in both legs (Figure 5B) (7). DNA was extracted from blood lymphocytes. No pathogenic variants were identified in a panel of 22 genes known to be associated with lymphatic problems. A diagnosis of WILD syndrome was made based on her clinical features. Genetic analysis is being performed on DNA from skin fibroblasts as part of an ongoing research study to identify the cause of WILD syndrome.

#### 2D histology.

H&E staining as well as staining for lymphatic vessel markers revealed no obvious vascular alteration (Figure 5C and Supplemental Figure 1, Q–T).

#### Whole-mount 3D histology.

A normal lymph vessel network with weak PROX1 expression was shown (Figure 5, D–H). A very low number of lymphatic valves was detectable. No cystic vascular lesions or dilated vessels were detected. Similar to case 4, Podoplanin-positive lymphatic vessels packed with red blood cells were observed (Figure 5D, white arrowheads), indicating that the telangiectasias represented lymphatic vessels containing blood, hence their red color.

### Blood-lymphatic vessel shunts can be detected in erythematous cutaneous telangiectasias

To further investigate the red color of the lymphatic vasculature in more detail, a thorough analysis of all optical sections from the light sheet image stacks for the presence of erythrocytes in lymphatic vessels was performed. In contrast with case 3 (Figure 3D), blood-filled lymphatic vessels were identified at multiple positions in cases 4 and 5 (Figure 4D and Figure 5D, white arrowheads) using autofluorescence of red blood cells. Following the blood-filled vessels in 3D space revealed a potential connection site between blood vessels and lymphatic vessels, resulting in blood-lymphatic shunting (Supplemental Figure 3) and therefore the presence of red blood cells in lymphatic vessels.

## Discussion

Cutaneous erythematous lesions resembling vascular nevi or birthmarks are generally assumed to be blood vascular in origin. Here we describe 5 erythematous cutaneous vascular malformations on legs of patients with primary lymphedema. Lesions in 2 cases were considered nevus flammeus, and lesions in the other 3 cases were clinically seen as erythematous telangiectasias. All lesions proved to be lymphatic vessels on histological analysis of biopsies.

Blood is frequently found in abnormal dermal lymphatic vessels and particularly malformations, e.g., lymphangioma circumscriptum (8). Blood vessels and lymphatic vessels have the same embryological origins, so, in vascular malformations, it may not be surprising if dermal vessels are not fully differentiated and may appear like each other (hybrid). In development, platelets are important for maintaining venous integrity, and so, in malformations, lymphatics can be connected to blood vessels, resulting in blood shunting from one vessel to the other (9, 10).

Port-wine birthmarks/nevus flammeus are always considered blood vascular in type. As Happle (1) states: “the term capillary malformation is presently used to designate numerous quite different disorders such as port-wine birthmark (nevus flammeus), the salmon patch, the vascular nevus of the ‘megalencephaly-capillary malformation syndrome’ (MCAP) and the skin lesions of other non-hereditary conditions such as ‘capillary malformation-arteriovenous malformation’ (CM-AVM) as well as hereditary traits such as autosomal recessive ‘microcephaly-capillary malformation’ (MICCAP)” (1). There is no mention of lymphatic origin for capillary malformations. The implication is that all capillary malformations are blood vessel in origin, but as demonstrated from results presented here, lymphatic capillary malformation should be added. Maari and Frieden in 2004 recognized that some port-wine birthmarks have a strong connection to associated lymphatic disease, but there was no histological evidence to support their statement (11).

The 3D histological data from the nevus flammeus presented here showed cystic lesions of a lymphatic malformation of the identified vessels. In contrast, erythematous cutaneous telangiectasias had singular distinct connection sites between blood and lymphatic vessels (in nevus flammeus no distinct connection sites could be detected). A general transition from malformed blood vessels to lymphatic malformations appears to be the most common pattern in the analyzed samples.

Telangiectasia simply means “end vessel dilatation” (from Greek: *telos* = end; *angeion* = vessel; *ektasis* = stretching out, extension, dilatation). Their spidery nature indicates vessels horizontal to the skin surface, e.g., spider telangiectasias. The redness is assumed to be from blood cells, and telangiectasias are considered to represent expansion of preexisting blood vessels. However, the erythematous cutaneous telangiectasias observed here proved to be of lymphatic phenotype on histological analysis.

There are reports of cutaneous capillary-lymphatic malformations (12). Net-like superficial lymphatic malformations have been described and equate to the telangiectatic lymphatic malformations described here. Noguera-Morel et al. described 3 examples of distinctive progressive, superficial red to purple patches composed of an arborizing network of vessels, histologically demonstrating anomalous lymphatics in the upper dermis. They suggest these cases are best considered as a distinct form of superficial lymphatic malformation (13). Vide et al. described 1 case of a lymphatic malformation in the upper dermis manifesting as transient purple reticulated patches, distinct from those included in the ISSVA classification and distinct from hobnail hemangioma (14). The third published case described red to purplish macules with a finely reticulated pattern of vascular structures. Dermoscopy showed arborizing telangiectatic vessels, and biopsy confirmed a lymphatic origin (15).

In all published cases, the telangiectasia lesions were not congenital and were often transient, remaining in place for a few weeks and then fading away slowly while others appeared in the same area. This was true for our cases of erythematous capillary-lymphatic malformations appearing as telangiectasias. Their behavior is similar to the reappearing “lymph blisters” seen on the skin surface with a lymphangioma circumscriptum (8). We believe that these erythematous capillary-lymphatic malformations may represent engorgement of dermal lymphatic vessels due to lymph reflux (dermal backflow) from a deeper lymphatic malformation, which may be associated with lymphedema. We hypothesize that as dermal intralymphatic pressures rise and fall, then the visible nature of these lesions comes and goes.

But why are these lesions red? Intralymphatic blood cells would be one answer, but red cells are not always found on biopsy. Dermal lymphatics can be red if inflamed (lymphangitis). Dermal lymphatics infiltrated by metastatic cancer (lymphangitis carcinomatosis) can present with a similar appearance. Under these circumstances red cells are not observed on biopsy, and so redness may be due to mechanisms other than luminal red cells (16). Nevertheless, blood-filled lymphatic vessels were identified at multiple positions in cases 4 and 5 using autofluorescence of red blood cells. Following the blood-filled vessels in 3D space revealed a distinct connection site between blood vessels and lymphatic vessels, resulting in blood-lymphatic shunting and therefore the presence of red blood cells in lymphatic vessels. However, this would need further investigation using relevant immunofluorescent markers.

**Figure 6:**
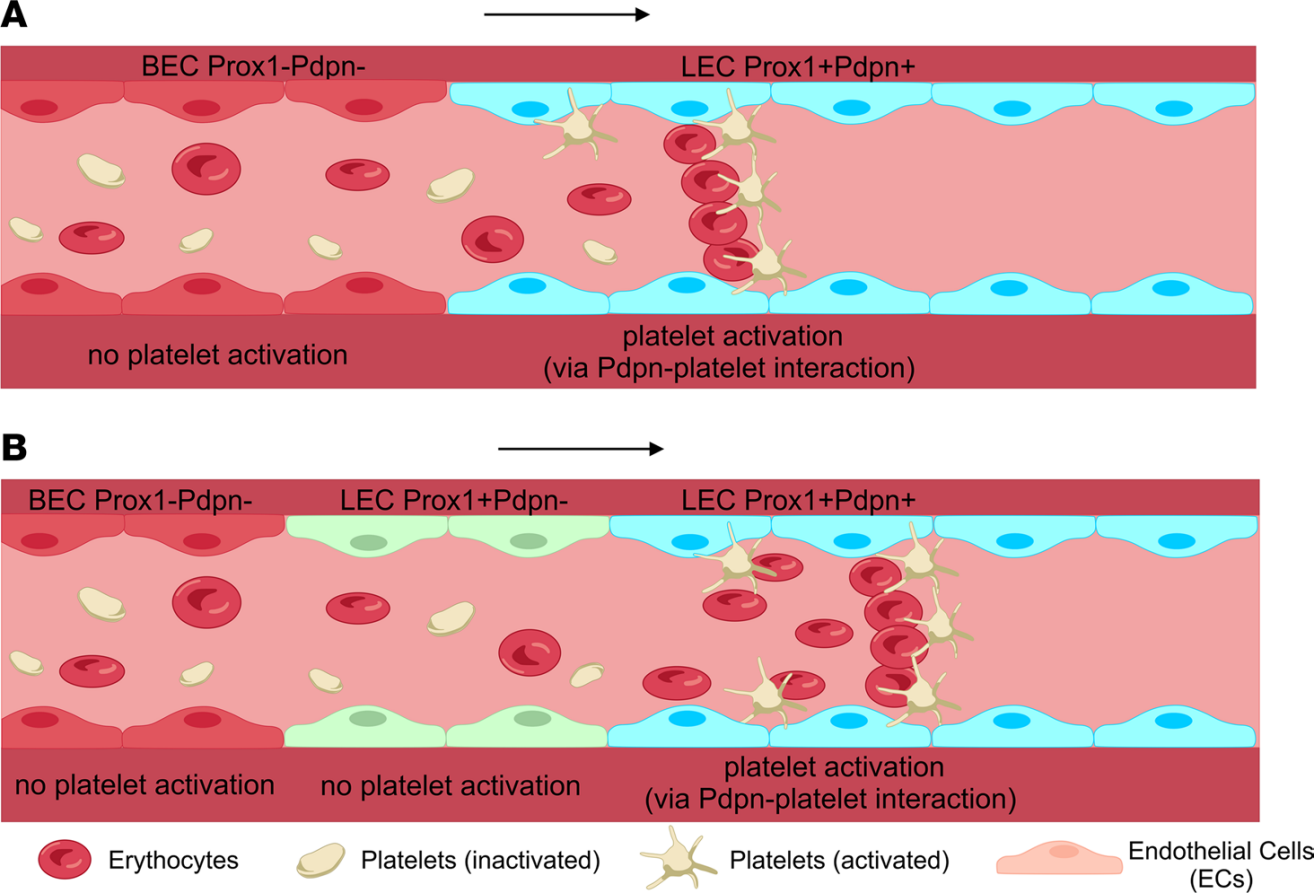
Schematic representation of hypothesized mechanism leading to blood-filled lymphatic vessels. (**A**) In contrast to blood endothelial cells (BECs), lymphatic endothelial cells (LECs) lining lymphatic vessels express the lymphatic markers PROX1 and Podoplanin (PDPN) (blue LECs, right). PDPN, a surface protein, binds platelets, resulting in their activation, which enables them to bind any red blood cells entering the lymphatic vessel. It is assumed that under normal physiological conditions if a shunt appears between a blood vessel and a lymphatic vessel in the skin, blood with all its components (including red blood cells and platelets) can escape into the lymphatic vessels. However, due to the immediate PDPN activation of the platelets, red blood cells will be bound, and filling of the lymphatic vessels with red blood cells is prevented. (**B**) In the hypothesized model, if a shunt appears between a blood vessel and a lymphatic vessel, which do not express PDPN (green LECs, middle), the platelets entering the lymphatics are not activated and therefore will not bind the entering red blood cells. This way blood filling of the lymphatic capillaries can happen, which make them appear as an erythematous cutaneous capillary malformation (nevus). Arrow, direction of flow of red blood cells/blood.

What the current study did demonstrate using lymphatic markers was an altered expression of PROX1 and Podoplanin in the malformed vessels. PROX1-positive vessels near blood vessels showed no, or weak, expression of Podoplanin, whereas more distant PROX1-positive vessels expressed Podoplanin. An important role of Podoplanin, expressed by lymphatic vessels, is in preventing postnatal blood filling of the lymphatic vascular system (17). This is a platelet-dependent process (18). Therefore, it is tempting to hypothesize that the altered expression of the lymphatic marker Podoplanin results in blood filling of lymphatic vessels, as Podoplanin signaling has been shown to be essential for platelet activation and separation of blood and lymphatic vessels (17, 18). Due to downregulation of Podoplanin on PROX1-positive lymphatic endothelial cells located next to blood endothelial cells, activation of platelets, while entering Podoplanin-negative lymphatic vessel structures, was impaired (Figure 6). This resulted in the presence of erythrocytes and white cells in lymphatic vessels. Our hypothesis is supported by studies showing dermal blood-lymphatic vascular shunting in Podoplanin-, Syk-, and Clec2-deficient mice (17–19).

From our cases reported here, and those in the literature, it is important to recognize that cutaneous erythematous vascular lesions could be lymphatic in origin. This would have important implications for making a correct diagnosis for phenotyping of patients and for genotyping if appropriate. Treatment with PIK3CA inhibitors or MAP kinase inhibitors might be appropriate if a somatic mutation is identified (20, 21). The importance of recognizing that these lesions could be lymphatic in origin might also have implications for infection risk, as lymphatic malformations have a higher incidence of infection (22).

In conclusion, erythematous skin lesions may not be blood vascular in origin. As demonstrated here, cutaneous erythematous capillary malformations can be of a lymphatic, not blood vascular, phenotype. Biopsy and 3D whole-mount investigation is necessary for the distinction between the two. The lymphatic cystic lesions, nonuniform expression of lymphatic vessel markers, and disconnected lymphatic network within the port-wine birthmarks suggest a malformation, whereas the erythematous telangiectasias seem to represent expanded but not necessarily malformed dermal lymphatic vessels. An erythematous capillary-lymphatic malformation should be considered in vascular anomalies where other lymphatic abnormalities such as lymphedema are present. Blood is the most likely explanation for the color, which might access the lymphatics through lympho-venous shunts or opening up of lympho-venous anastomoses.

## Methods

### Recruitment and biopsy.

Two patients with cutaneous erythematous vascular “nevus flammeus” lesions and lower limb primary lymphedema and 3 patients with erythematous telangiectasia and limb lymphedema were recruited for skin biopsy and histological analysis from 2 national primary lymphedema clinics in the United Kingdom (Derby and London). Under local anesthetic, 6 mm punch biopsies were obtained. Besides standard 2D histology, 3D histological analysis was performed using light sheet imaging: Lightsheet 7 (Zeiss) and Ultramicroscope II (LaVision BioTec).

### Genetic testing.

Diagnostic genetic testing in our clinic was performed according to the clinical presentation. For patients with segmental overgrowth and vascular malformations (cases 1 and 2), a skin biopsy of an affected area was obtained, DNA was extracted, and samples were screened for postzygotic, mosaic mutations on the overgrowth panel (includes genes in the AKT and RAS/MAP kinase pathway) as per standard protocol in the SW Thames Regional Centre for Genomics.

For patients in whom we suspected a germline mutation, we took blood for the lymphedema gene panel or whole-genome sequencing. The current list of genes on the Genomics England Primary Lymphoedema gene panel (Version 3.2) can be viewed here: https://panelapp.genomicsengland.co.uk/panels/65/

### Antibodies.

The following antibodies were used: mouse monoclonal IgG_1_ anti-human Podoplanin (MA1-83884, Invitrogen), rabbit polyclonal IgG anti-human PROX-1 (102-PA32AG, ReliaTech), donkey polyclonal anti-mouse IgG Alexa Fluor 568 (A10037, Life Technologies), and donkey polyclonal anti-rabbit IgG Alexa Fluor 488 (A21206, Life Technologies).

### Standard immunofluorescence histology.

Tissue sectioning of tissue samples was performed as described before (4). After fixation of skin biopsies in 4% paraformaldehyde/phosphate-buffered saline (PFA/PBS) for 4 hours, samples were washed in PBS, embedded, and snap-frozen in OCT. Then 10 μm cryosections were generated. Cryosections were incubated in ice-cold methanol for 15 minutes, washed, and blocked (10% chicken serum, 0.3% Triton X-100 in PBS). Following blocking, tissue sections were incubated for 1 hour with primary antibodies (diluted in 1% BSA, 1% chicken serum, 0.3% Triton X-100 in PBS), washed thrice in PBS-T (0.1% Tween 20 in PBS), and finally incubated in Alexa dye–conjugated secondary antibodies (see *Antibodies*). After sample mounting in Mowiol (Calbiochem), samples were imaged using a Zeiss LSM 980 confocal microscope (25× oil, NA = 0.8).

### Standard histology.

Histochemical staining was performed on 5 μm sections. A Ventana BenchMark ULTRA platform was used (Roche).

### Whole-mount skin biopsy immunofluorescence staining for light sheet microscopy.

Fresh skin biopsies were fixed in 4% PFA/PBS for 4 hours at 4°C. Samples were permeabilized (0.5% Triton X-100/PBS), then blocked in PermBlock solution (1% BSA, 0.5% Tween 20 in PBS), and whole-mount immunofluorescence staining was performed using indicated primary antibodies and Alexa dye–coupled secondary antibodies diluted in PermBlock solution. Following each staining step, samples were washed thrice in PBS-T (3, 4).

For 3D histological analysis, the entire sample was subjected to whole-mount immunofluorescence staining for the lymphatic markers PROX1 and Podoplanin to detect all the lymphatic vasculature within the specimen.

### Optical clearing of whole-mount stained skin biopsies.

Optical clearing of skin samples was performed as described before (3, 4). Briefly, whole-mount immunofluorescence-stained skin biopsies were embedded in 1% low-melting-point agarose (Thermo Fisher Scientific) and dehydrated in increasing methanol concentrations (50%, 70%, 95%, >99.0%, >99.0% [v/v] methanol, each step 30 minutes). After incubation in a benzyl alcohol/benzyl benzoate (BABB; MilliporeSigma) (ratio 1:2 [v/v])/methanol (>99.0% [v/v]) mixture for 4 hours, samples were incubated in BABB for 4 hours twice. Optically cleared skin biopsies were stored in BABB for imaging.

### Light sheet microscopy, 3D reconstruction, and data analysis.

Immunofluorescence-stained and optically cleared skin biopsies were optically sectioned using a LaVision UltraMicroscope II (LaVision BioTec). Image stacks were captured with a step size of 1 μm and at various magnifications. Following imaging, optical sections (>2,000 single optical 2D sections) were digitally 3D reconstructed and analyzed. Digital 3D reconstruction of light sheet image stacks was performed using Imaris Microscopy Image Analysis Software (Oxford Instruments) (3, 5).

### Study approval.

Ethical approval was obtained from the local health research authority (London, United Kingdom) (REC reference number 12/LO/0498). The study has been conducted according to the principles expressed in the Declaration of Helsinki. All patients provided their written informed consent to the study. All patients provided written informed consent for the use of their photographs.

### Data availability.

The data that support the findings of this study are available from the corresponding authors upon reasonable request.

## Author contributions

Conceptualization and methodology were contributed by RH, MVZ, PO, and PSM; RH, MVZ, RYB, SU, and NRH performed experiments; RH, MVZ, BM, and RYB performed data analysis and data curation; BH, BM, and CW performed sample collection; MVZ, KG, SM, VK, and KR provided patients; RH, MVZ, KG, SM, PO, and PSM provided manuscript preparation; RH, RYB, and SU provided figure preparation and visualization; RH, PO, and PSM provided project supervision; RH, KG, SM, PO, and PSM acquired funding; and all authors have read and agreed to the published version of the manuscript. The order of the co–first authors in the author list was decided using the COPE Discussion Document, 2014.

## Supplementary Material

Supplemental data

## Figures and Tables

**Table 1:**
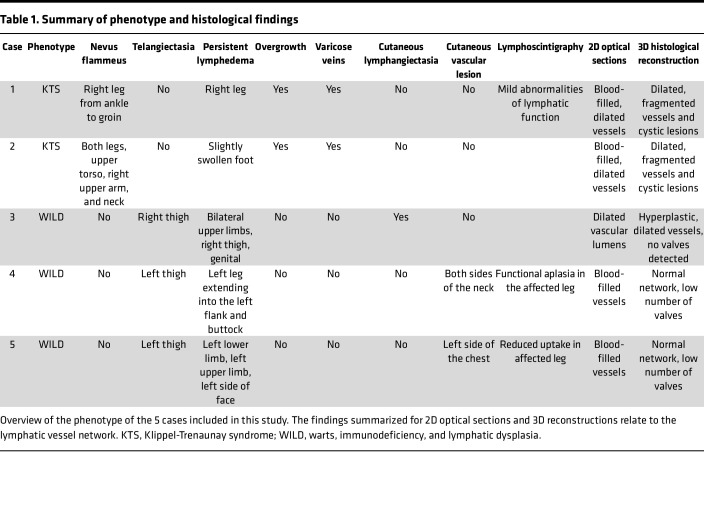
Summary of phenotype and histological findings

## Version 1 09/12/2023

In-Press Preview

## Version 2 10/23/2023

Electronic publication
