# Supplementary material for: Erythematous capillary-lymphatic malformations mimicking blood vascular anomalies
Source: JCI Insight. 2023 Oct 23;8(20):e172179. doi: 10.1172/jci.insight.172179 (PMC10619487; doi:10.1172/jci.insight.172179)
Supplement: Supplemental data [file jciinsight-8-172179-s208.pdf]

Hägerling et al.

SUPPLEMENTAL DATA:

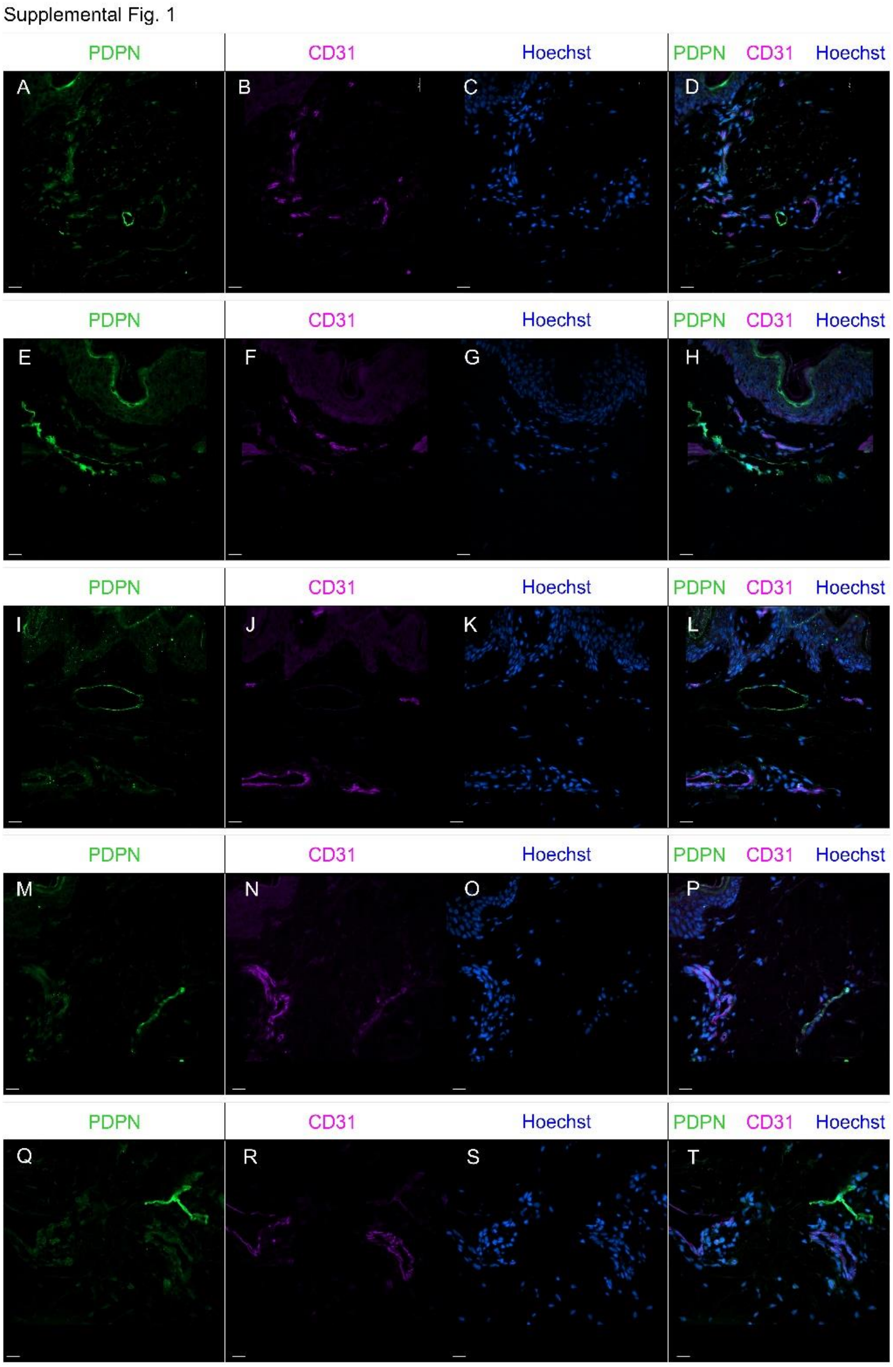

Supplemental Figure 1: Immunofluorescence staining for lymphatic vessels in histological sections from biopsies from cases 1-5. Immunofluorescence staining of histological sections for indicated blood (CD31) and lymphatic vessel (PDPN) markers are shown. Nuclei staining with Hoechst. Scale bar = 100 µm.

Supplemental Fig. 2

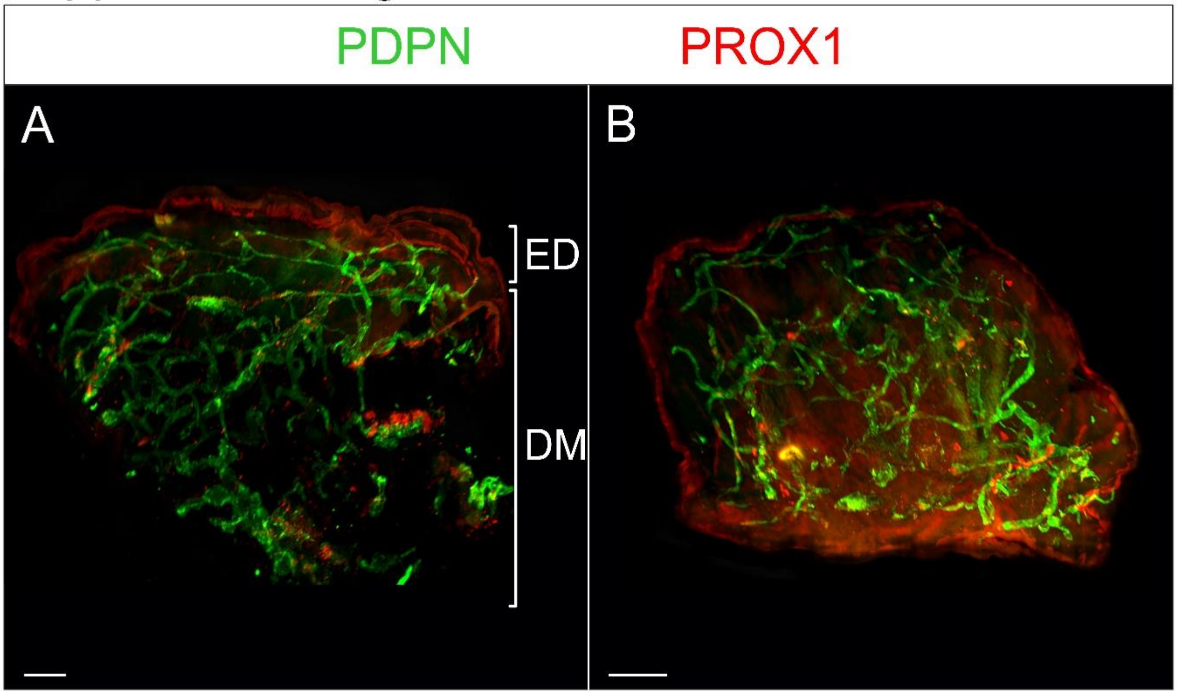

Supplemental Figure 2: Three-dimensional (3D) reconstruction of the dermal lymphatic vasculature in healthy control. Shown are maximum intensity projections of 3D reconstructed lymphatic vasculature of wholemount immunostained skin biopsies from a healthy individual imaged using light sheet microscopy. Podoplanin (PDPN) served as a lymphatic endothelial cell-membrane marker, the transcription factor PROX1 as lymphatic endothelial nuclei marker. Detected antigens and respective colours are indicated. (A) Visualisation of the tissue volume with the epidermis (ED) apically followed by the papillary dermis located at top and cutaneous plexus at bottom of dermis (DM). (B) Digitally rotated view of the same specimen, showing the vessels of the papillary plexus viewed en face through the epidermis. Scale bars: 200 µm.

Supplemental Fig. 3

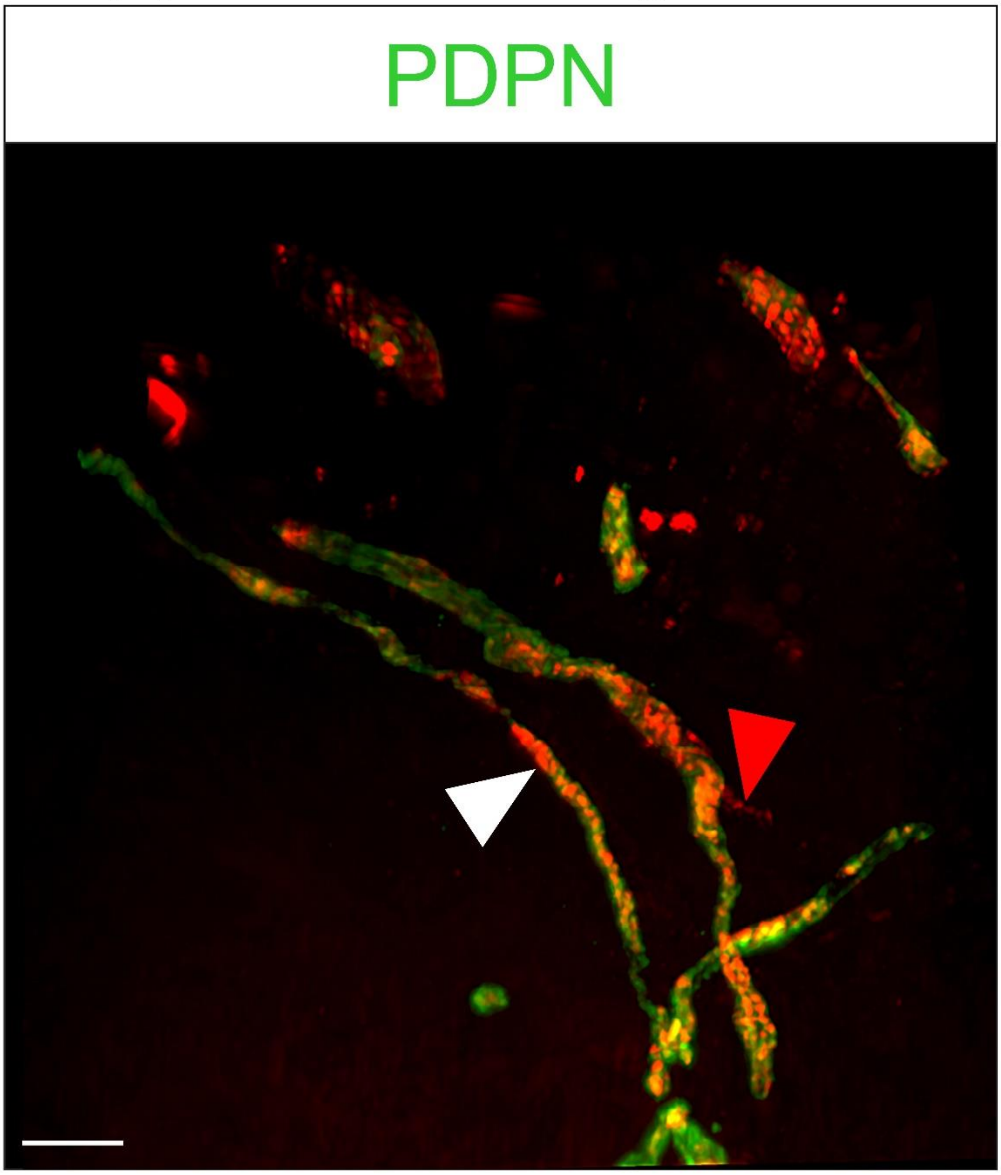

Supplemental Figure 3: Blood-filled lymphatic vessels and potential connection site between blood and lymphatic vasculature. Maximum intensity projections of 3D reconstructed lymphatic vasculature and presence of red blood cells within lymphatic vascular lumen (white arrowhead). Connection site between blood and lymphatic vessels highlighted by stream of erythrocytes (red arrowhead) draining into Podoplanin-positive lymphatic vessels (green). Visualization of the erythrocytes using autofluorescence (red). Scale bar = 100 µm.
